# Current clinical practice and outcome of neoadjuvant chemotherapy for early breast cancer: analysis of individual data from 94,638 patients treated in 55 breast cancer centers

**DOI:** 10.1007/s00432-022-03938-x

**Published:** 2022-04-05

**Authors:** O. Ortmann, J.-U. Blohmer, N. T. Sibert, S. Brucker, W. Janni, A. Wöckel, A. Scharl, S. Dieng, J. Ferencz, E. C. Inwald, S. Wesselmann, C. Kowalski, for 55 breast cancer centers certified by the German Cancer Society, E. Denisjuk, R. Csorba, D. Rezek, S. Peschel, D. Denschlag, F. Schad, D. Dieterle, B. Lex, L. Rieger, F. Flock, A. Cramer, H.-J. Strittmatter, J. Bettscheider, C. Lindner, A. Stefek, W. Pauker, A. Hönig, M. Schrauder, D.Seeger, A. Kleine-Tebbe, A. Bleimehl, U. Groh, G. Gebauer, H.-W. Vollert, A. Zorr, W. Friedmann, P. Krabisch, S. Fritz, A. Schwämmle, W. Lucke, S. Schmatloch, W. Heyl, P. Deuschle, M. Arfsten, P. Scheler, A. Bleimehl, A. Ruf-Dördelmann, B. Brückner, L. Bauer, M. Koch, J. Monner, A. Nixdorf, A. Merte, F. Beldermann, J.-U. Blohmer, W. Janni, R. Felberbaum, R. L. De Wilde, P. Bolkenius, A. Lebrecht, O. Ortmann, E.-F. Solomayer, S. Hartmann, A. Hartkopf

**Affiliations:** 1 Department of Gynecology and Obstetrics, University Medical Center, Landshuter Straße 65, 93053 Regensburg, Germany; 2 Department of Gynecology and Breast Center, Charité-Universitätsmedizin, Berlin, Germany; 3 German Cancer Society, Berlin, Germany; 4 Department of Women’s Health, University of Tübingen, Tübingen, Germany; 5 Department of Gynecology, University of Ulm, Ulm, Germany; 6 Department of Obstetrics and Gynecology, University Hospital of Würzburg, Würzburg, Germany; 7 Frauenklinik, Klinikum St. Marien Amberg, Amberg, Germany; 8 OnkoZert, Neu-Ulm, Germany

**Keywords:** Early breast cancer, Neoadjuvant chemotherapy, Breast cancer centers, Guidelines, Oncobox research

## Abstract

Neoadjuvant chemotherapy (NACT) is frequently used in patients with early breast cancer. Randomized controlled trials have demonstrated similar survival after NACT or adjuvant chemotherapy (ACT). However, certain subtypes may benefit more when NACT contains regimes leading to high rates of pathologic complete response (pCR) rates. In this study we analyzed data using the OncoBox research from 94,638 patients treated in 55 breast cancer centers to describe the current clinical practice of and outcomes after NACT under routine conditions. These data were compared to patients treated with ACT. 40% of all patients received chemotherapy. The use of NACT increased over time from 5% in 2007 up to 17.3% in 2016. The proportion of patients receiving NACT varied by subtype. It was low in patients with HR-positive/HER2-negative breast cancer (5.8%). However, 31.8% of patients with triple-negative, 31.9% with HR-negative/HER2-positive, and 26.5% with HR-positive/HER2-positive breast cancer received NACT. The rates of pCR were higher in patients with HR-positive/HER2-positive, HR-negative/HER2-positive and triple-negative tumors (36, 53 and 38%) compared to HR-positive/HER2-negative tumors (12%). PCR was achieved more often in HER2-positive and triple-negative tumors over time.

This is the largest study on use and effects of NACT in German breast cancer centers. It demonstrates the increased use of NACT based on recommendations in current clinical guidelines. An improvement of pCR was shown in particular in HER2-positive and triple-negative breast cancer, which is consistent with data from randomized controlled trails.

## Introduction

Neoadjuvant chemotherapy (NACT) for breast cancer was initially introduced to treat locally advanced disease to make it more accessible for surgery. Also, it became popular to reduce the size of large tumors to allow breast-conserving surgery (BCS). An additional benefit of NACT is the option to reduce morbidity caused by surgery in patients with histologically proved metastatic axillary lymph nodes (N1) and to allow targeted axillary dissection (TAD, i.e., excision of the biopsied and clip marked lymph node in combination with sentinel node excision) in case of pathologic complete remission (pCR) of lymph node metastasis (Caudle et al. 2016). NACT is widely accepted as an in vivo test for chemosensitivity (Houssami et al. 2012; Minckwitz et al. 2011). A pCR is a surrogate marker for better disease free (DFS) and overall survival (OS) (Cortazar et al. 2014). A meta-analysis which compared outcome data of randomized trials initiated between 1983 and 2002 compared NACT and adjuvant chemotherapy (ACT). There were no differences in breast cancer mortality and OS but an increase in local recurrences in patients receiving NACT (Early Breast Cancer Trialists’ Collaborative Group (EBCTCG) 2018). This meta-analysis must be interpreted with caution. Only 902 of the included 4756 women received anthracyclins and taxanes, no patient was treated with trastuzumab, and no data for therapy monitoring or surgical planning were available, for instance. Patient-level information about axillary surgery and radiotherapy were not available. The concept of NACT was used to optimize systemic treatment with the goal to increase survival rates. It was claimed that treatment choice depending on molecular subtypes of the disease or the in vivo sensitivity observed during NACT could lead to better outcomes with the improvement of OS. In randomized controlled trials it was demonstrated that this assumption proved to be correct when using pCR as an outcome parameter which correlates with OS (Cortazar et al. 2014). In particular, NACT was effective in disease with more aggressive subtypes such as triple-negative, HER2-positive and high-grade breast cancer whereas steroid hormone receptor (HR) positive tumors responded weaker. Recently, pCR rates could be substantially improved by using NACT in combination with the anti-HER2 antibodies trastuzumab and pertuzumab by up to 60% (Loibl et al. 2017). Furthermore, it has been shown that patients without pCR had a benefit of post-neoadjuvant treatment, e.g., in HER2-positive breast cancer with TDM-1 (Minckwitz et al. 2019).

These developments led to the introduction of NACT into routine care of patients with early breast cancer. However, information about the current clinical practice and its oncological outcome is sparse. We therefore conducted a study including individual level quality assurance data from 94,638 patients with early breast cancer treated in 55 breast cancer centers certified by the German Cancer Society (DKG) and the German Society of Senology. The changes of NACT use and the relationship between NACT/ACT use over time, patient and center characteristics were described during the years from 2007 to 2018. In addition, associations between NACT and pCR rate were analyzed in different breast cancer subtypes.

## Methods

### Data

We used data routinely collected for quality assurance purposes (certification, clinical cancer registries) in breast cancer centers certified according to the criteria of the German Cancer Society and the German Society of Senology (Kowalski et al. 2015). Patient data are stored locally in the hospitals containing identical information in varying formats according to the locally used software. To harmonize the data, the software tool OncoBox with the specification for breast cancer was used locally. The OncoBox formats the data into an xml dataset with individual information being de-identified for use outside the center. Datasets contain information on age, diagnosis (e.g., TNM, tumor localization), treatment (e.g., type of surgery, systemic therapy), and outcomes. All centers certified at that time were asked in spring 2019 to participate to analyze patterns of care and variation between centers and over time using these routinely collected data. No formal ethical review board (ERB) statement was necessary after consultation with the University of Regensburg ERB. Fifty-five centers participated and transferred data.

Patients were included in the analytical sample when they received surgery for early breast cancer (confirmed diagnosis of ICD-code C50.) between 2007 and 2018, aged 18 or older, who had a gender assigned, and who had no metastases (M0) at diagnosis. For patients who had more than one case reported (if they had synchronous or asynchronous bilateral disease) only one reported case was considered to allow for the independence of observations.

### Variables

The dependent variable was use of chemotherapy (CHT) with the four responses NACT, NACT plus post-neoadjuvant chemotherapy (ACT), ACT, or no CHT. Patients were considered receiving CHT if they had a tumor board recommendation for CHT and/or a start and/or termination date of CHT related to surgery. For the analyses, the variable was split into the two variables NACT (including NACT plus post-neoadjuvant chemotherapy) vs. no NACT and, for the remaining patients, ACT (excluding NACT plus post-neoadjuvant chemotherapy) vs. no ACT.

Independent patient level variables included in the analyses were age in years at diagnosis (continuous), gender (male/female), year of diagnosis (continuous), T (T0, TIS/DCIS, T1, T2, T3, T4) and N (N0, N1, N2, N3, N4), staging, grading (G1, G2, G3, G4), type of surgery (mastectomy, BCS, BCS followed by mastectomy), and tumor subtype (hormone receptor (HR) negative/HER2 negative, HR negative/HER2 positive, HR positive/HER2 negative, HR positive/HER2 positive). Histologic type of tumor and tumor grading were determined by pathology examination of biopsies taken before surgery since this is relevant for the decision on NACT. For patients without NACT and missing information on T or N, the pathological information was used. Patients with no information on T or N staging, grading and subtype were excluded from the analytic dataset, but included in sensitivity analyses.

Center variables investigated included teaching status (university hospital vs. not), annual primary case number in 2018 (continuous), ownership (private, charitable, public) and urbanity of center location 100,000 or less vs. more than 100,000).

### Statistical analyses

Data were analyzed descriptively presenting relative and absolute frequencies of sample characteristics according to CHT (Table 1). In a second step, generalized linear mixed-effects models were estimated to take the hierarchical structure of patients (level 1) treated in centers (level 2) into account. In model 1, NACT vs. no NACT was predicted. In model 2, ACT vs. no ACT was predicted for patients not receiving NACT previously. For both models, we first estimated null models that included no predictor variables to receive null model intraclass correlation coefficients (ICC). Higher ICCs (range from 0–1) indicate a higher similarity of units within the same group, in this case breast cancer centers. ICCs close to 0 on the contrary indicate little variance across centers, in other words, little variation in treatment patterns across centers. Models 1 and 2 included all patient variables and random center effects. Odds ratios (OR) are presented with 95% confidence intervals (CI). In additional analyses we included the center characteristics as level 2 variables (Appendix, Tables 6, 7). Since centers started documentation at different time points and thus not all centers had data for earlier years when NACT was less common, we expected these analyses to result in high variation between centers (interaction of time and center). We therefore re-ran all analyzes on a year-by-year basis, not only including patient but also center characteristics in sensitivity analyses (available upon request). Patients with missing information on staging, grading and subtype were excluded from the main analyses but included in sensitivity analyses in which a separate effect for missing information was estimated (Appendix, Tables 8, 9). All statistical analyses were performed using R version 4.0.2. A *p* value < 0.05 was considered statistically significant.

**Table 1:** Characteristics of patients treated with NACT, ACT or no CHT

Characteristic	No chemotherapy, *N* = 56,753^1^	NACT, *N* = 10,372^1^	ACT, *N* = 27,107^1^	NACT + ACT,*N* = 406^1^
Age	66 (5676)	52 (4461)	58 (4966)	50 (4260)
Sex				
Female	56,368 (60%)	10,346 (11%)	26,920 (29%)	406 (0.4%)
Male	385 (64%)	26 (4.3%)	187 (31%)	0 (0%)
Year of diagnosis				
2007	2285 (55%)	202 (4.9%)	1631 (40%)	6 (0.1%)
2008	3260 (55%)	339 (5.8%)	2286 (39%)	9 (0.2%)
2009	3923 (57%)	380 (5.5%)	2621 (38%)	11 (0.2%)
2010	4100 (57%)	410 (5.7%)	2660 (37%)	6 (< 0.1%)
2011	4268 (58%)	522 (7.1%)	2559 (35%)	19 (0.3%)
2012	5155 (60%)	691 (8.0%)	2718 (32%)	26 (0.3%)
2013	5301 (62%)	750 (8.7%)	2523 (29%)	40 (0.5%)
2014	5419 (61%)	960 (11%)	2361 (27%)	75 (0.9%)
2015	5499 (62%)	1204 (13%)	2172 (24%)	49 (0.5%)
2016	5853 (62%)	1593 (17%)	1956 (21%)	38 (0.4%)
2017	5683 (61%)	1716 (18%)	1918 (20%)	50 (0.5%)
2018	6007 (64%)	1605 (17%)	1702 (18%)	77 (0.8%)
Tumor size				
T0	117 (66%)	11 (6.2%)	47 (27%)	1 (0.6%)
TIS	634 (74%)	10 (1.2%)	208 (24%)	0 (0%)
T1	27,520 (68%)	2766 (6.8%)	10,113 (25%)	93 (0.2%)
T2	12,313 (46%)	5377 (20%)	8780 (33%)	217 (0.8%)
T3	1339 (41%)	856 (26%)	1010 (31%)	39 (1.2%)
T4	1441 (52%)	825 (30%)	487 (17%)	37 (1.3%)
Missing	13,389 (66%)	527 (2.6%)	6462 (32%)	19 (< 0.1%)
Nodal stage				
N0	47,121 (69%)	5330 (7.8%)	15,916 (23%)	200 (0.3%)
N1	7144 (37%)	4262 (22%)	7957 (41%)	161 (0.8%)
N2	1165 (31%)	549 (15%)	2032 (54%)	18 (0.5%)
N3	632 (33%)	169 (8.9%)	1100 (58%)	7 (0.4%)
Missing	691 (79%)	62 (7.1%)	102 (12%)	20 (2.3%)
Grading				
G1	11,541 (90%)	68 (0.5%)	1151 (9.0%)	5 (< 0.1%)
G2	36,539 (69%)	1819 (3.4%)	14,649 (28%)	111 (0.2%)
G3	7367 (35%)	2619 (12%)	10,839 (51%)	276 (1.3%)
G4	28 (65%)	4 (9.3%)	11 (26%)	0 (0%)
Missing	1278 (17%)	5862 (77%)	457 (6.0%)	14 (0.2%)
Type of surgery				
BCS	42,273 (62%)	6948 (10%)	18,981 (28%)	233 (0.3%)
MAS	11,759 (57%)	2984 (14%)	5755 (28%)	150 (0.7%)
BCS-MAS	2721 (49%)	440 (7.9%)	2371 (43%)	23 (0.4%)
Subtype				
HR + /HER2−	48,578 (70%)	3962 (5.7%)	16,883 (24%)	99 (0.1%)
HR−/HER2−	2497 (26%)	2915 (30%)	4136 (43%)	183 (1.9%)
HR + /HER2 +	2360 (28%)	2116 (26%)	3728 (45%)	77 (0.9%)
HR−/HER2 +	937 (24%)	1216 (31%)	1737 (44%)	37 (0.9%)
Missing	2381 (75%)	163 (5.1%)	623 (20%)	10 (0.3%)

*ACT* adjuvant chemotherapy, *NACT* neoadjuvant chemotherapy, *NACT + ACT* neoadjuvant chemotherapy plus adjuvant chemotherapy, *BCS* breast-conserving surgery, *MAS* mastectomy, *BCS-MAS* breast-conserving surgery followed by mastectomy

## Results

**Fig. 1:**
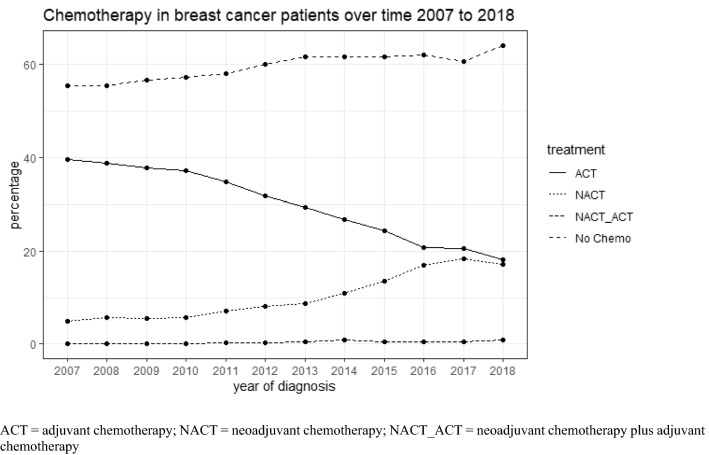
Time-dependent use of NACT and adjuvant treatments. *ACT* adjuvant chemotherapy, *NACT* neoadjuvant chemotherapy, NACT_ACT neoadjuvant chemotherapy plus adjuvant chemotherapy

The participating centers had a mean case number of 233 patients with a first diagnosis of breast cancer in 2018 (interquartile range 154–269); seven centers were university hospitals, 48 were not; 29 centers were located in municipalities of up to 100,000 inhabitants, 26 in those with more than 100,000 inhabitants. Table 1 presents the clinical characteristics of the analytical sample according to CHT use. Overall, in the records from 37,885 out of 94,638 (40.0%) patients CHT was documented, with 10,372 for NACT, 27,107 for ACT, and 406 for both. The rate of NACT increased from 5% in 2007 to 17.3% in 2016 and remained stable in 2017 and 2018 while NACT use increased ACT use decreased over time (Fig. 1, Table 1). Mean age of the patients treated with NACT was 52 years whereas it was 66 years in patients receiving no CHT. The sample included 598 male patients. The percentage of men receiving NACT was 4.3%, whereas in women it was 11.4%. In the whole population patients with larger tumors, higher tumor grading and positive lymph nodes were treated more often with NACT in the bivariate analyses (Table 1).

**Fig. 2:**
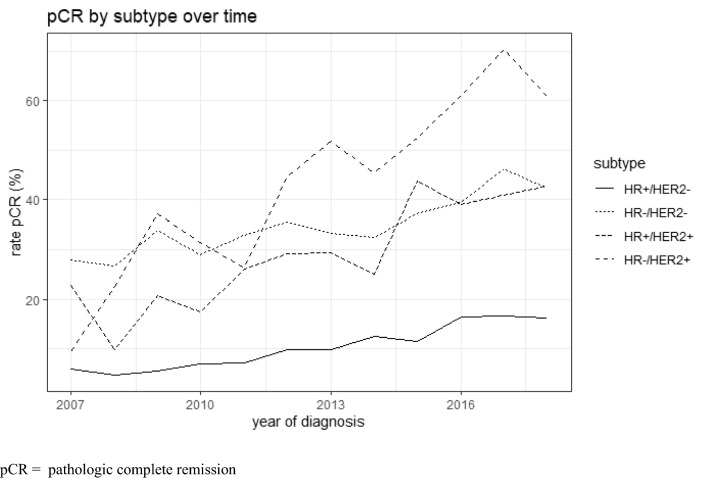
pCR by subtype over time, *pCR* pathologic complete remission

Regarding the different subtypes of breast cancer, patients with HER2-positive and triple-negative disease were treated more often with NACT. In total 31.8% of patients with triple-negative breast cancer received NACT or NACT + ACT. HR-positive/HER2-positive breast cancer patients were treated with NACT in 26.5% and HR-negative/HER2-positive patients in 31.9% (Table 2). For patients who received NACT we calculated proportions of patients for whom pCR was documented. The rates of pCR were higher in patients with HR-positive/HER2-positive, HR-negative/HER2-positive and triple-negative tumors (36, 53 and 38%) compared to HR-positive/HER2-negative tumors (12%) (Table 3). Furthermore, pCR was achieved more often in HER2-positive and triple-negative tumors over time (Fig. 2).

**Table 2:** Use of NACT in different subtypes of breast cancer

Characteristic	HR + /HER2− *N* = 69,522	HR−/HER2− *N* = 9731	HR + /HER2 + *N* = 8281	HR−/HER2 + *N* = 3927
No NACT	65,461 (94.2%)	6633 (68.2%)	6088 (73.5%)	2674 (68.1%)
NACT	4061 (5.8%)	3098 (31.8%)	2193 (26.5%)	1253 (31.9%)

*NACT* neoadjuvant chemotherapy

**Table 3:** pCR rates after NACT in different subtypes of breast cancer

Characteristic	HR + /HER2− *N* = 3978	HR−/HER2− *N* = 3046	HR + /HER2 + *N* = 2150	HR−/HER2 + *N* = 1230
No pCR	3498 (88%)	1879 (62%)	1386 (64%)	575 (47%)
pCR	480 (12%)	1167 (38%)	764 (36%)	655 (53%)

*N* 10,404, patients without information on pCR and subtype excluded, *NACT* neoadjuvant chemotherapy, *pCR* pathologic complete remission

After the exclusion of patients with missing information on any of the clinical characteristics T, N, G, and subtype, data from 65,667 patients with early breast cancer diagnosed between 2007 and 2018 were analyzed in generalized linear mixed-effects models. Models confirm the bivariate findings with higher odds of NACT compared to non-NACT with younger age, female gender, increasing T, N1/N2/N3 vs. N0, a higher grading, except for G4 (only *n* = 43 in the analytic sample), and triple negative or HER2-positive tumors (Table 4). Only for type of surgical therapy, the direction of the association changed in the multivariable model, with higher odds of NACT with BCS.

**Table 4:** Dependent variable NACT vs no NACT (Model 1)

	Odds ratio	95% CI-lower	95% CI-upper
(Intercept)	0.01	0.00	0.08
Age (cont.)	0.93	0.93	0.94
Male (ref. female)	0.67	0.33	1.36
Year of diagnosis			
2007 (ref.)	1		
2008	1.38	0.91	2.08
2009	1.28	0.85	1.92
2010	1.37	0.92	2.06
2011	1.76	1.19	2.62
2012	1.93	1.32	2.82
2013	1.87	1.28	2.71
2014	1.87	1.29	2.70
2015	3.02	2.11	4.33
2016	4.68	3.28	6.70
2017	3.93	2.76	5.60
2018	3.40	2.38	4.86
Tumor size			
T0	1.17	0.36	3.82
TIS/DCIS	0.08	0.03	0.23
T1 (ref.)	1		
T2	2.70	2.43	2.99
T3	3.66	2.99	4.48
T4	6.31	5.11	7.80
Nodal status			
N0 (ref.)	1		
N1	3.28	2.95	3.65
N2	3.02	2.30	3.96
N3	2.28	1.51	3.45
Grading			
G1	0.25	0.19	0.33
G2 (ref.)	1		
G3	2.09	1.89	2.32
G4	0.70	0.16	3.08
Type of surgery			
MAS	0.72	0.65	0.81
BCS (ref.)	1		
BCS-MAS	0.41	0.32	0.51
Subtype			
HR + /HER− (ref.)	1		
HR−/HER−	4.07	3.58	4.64
HR + /HER +	4.12	3.62	4.68
HR−/HER +	4.59	3.86	5.46

Model	*N* patient	*N* center	AIC	BIC	loglik	deviance	df.resid	ICC (cond.)
Model diagnostics								
Nullmodel	65,667	55	18,919.2	18,937.4	− 9457.6	18,915.2	65,665	0.920
Model 1	65,667	55	12,602.9	12,884.8	− 6270.4	12,540.9	65,636	0.850

*BCS* breast-conserving surgery, *MAS* mastectomy, *BCS-MAS* breast-conserving surgery followed by mastectomy, *cont* continuous, *ref* referent, *AIC* Akaike information criterion, *BIC* Bayesian information criterion, *loglik* log-likelihood, *df.resid* degrees of freedom residuals, *ICC* intraclass correlation coefficient

The high intraclass correlation coefficient (ICC) suggests that NACT is highly dependent on the center in which a patient is treated. However, none of the center effects urbanity, teaching status, ownership, and case number included in an additional model were significantly associated with NACT (Appendix, Table 6). The model fit was not superior to the model without center characteristics. Additional year-by-year analyses including estimates for urbanity, ownership, teaching status, and case number yielded similar results for the patient characteristics, while none of the center characteristics were statistically significant at *p* < 0.05 (available upon request). Due to missing information, especially regarding tumor size, we ran additional sensitivity analyses with separate estimates for missing information (Appendix, Table 8). Estimates were mostly similar in direction and strengths, except for the gender effect. Estimates also varied with regard to year of diagnosis, suggesting a learning curve in documentation over time. Lowest odds were found for the missing categories, suggesting a general poorer documentation for these patients (e.g., patients with no documented T stage also do not have documentation/information regarding CHT).

For patients without NACT, we then estimated generalized linear mixed-effects models to predict ACT over non-ACT use (Table 5). ACT use decreased with age and was more prevalent in male patients. Compared to 2007, it decreased from 2011 onward, increased with tumor size (except T4), and was more prevalent in node-positive patients and with higher grading, in patients receiving BCS followed by mastectomy compared to BCS alone and less prevalent in mastectomy alone, and in patients with another subtype than HR + /HER2−. Again, a sensitivity analysis was run including estimates for missing information (Appendix, Table 9) that yielded very similar estimates without having a better model fit. After adding center characteristics to the model fit, no relevant changes were found for the patient estimates, but patients treated in centers based in cities with more than 100,000 inhabitants had lower odds of receiving ACT. The model fit however was not superior compared to the model without center characteristics (Appendix, Table 7).

**Table 5:** Dependent variable ACT vs no CHT (Model 2)

	Odds ratio	95% CI-lower	95% CI-upper
(Intercept)	25.46	19.73	32.85
Age (cont.)	0.93	0.93	0.93
Male (ref. female)	1.83	1.42	2.36
Year of diagnosis			
2007 (ref.)	1		
2008	1.00	0.88	1.15
2009	1.05	0.93	1.19
2010	0.90	0.80	1.03
2011	0.79	0.69	0.89
2012	0.67	0.59	0.76
2013	0.61	0.54	0.69
2014	0.60	0.53	0.68
2015	0.54	0.48	0.61
2016	0.49	0.43	0.55
2017	0.54	0.48	0.61
2018	0.44	0.39	0.50
Tumor size			
T0	0.96	0.63	1.48
TIS/DCIS	0.59	0.48	0.73
T1 (ref.)	1		
T2	1.68	1.60	1.77
T3	1.48	1.31	1.66
T4	0.73	0.63	0.84
Nodal status			
N0 (ref.)	1		
N1	3.50	3.31	3.70
N2	5.11	4.54	5.75
N3	4.83	4.11	5.67
Grading			
G1	0.28	0.26	0.30
G2 (ref.)	1		
G3	2.78	2.63	2.93
G4	0.58	0.18	1.86
Type of surgery			
MAS	0.72	0.68	0.77
BCS (ref.)	1		
BCS-MAS	1.26	1.15	1.37
Subtype			
HR + /HER − (ref.)	1		
HR −/HER −	3.10	2.86	3.37
HR + /HER +	3.73	3.45	4.03
HR −/HER +	3.54	3.14	3.98

Model	*N* patient	*N* center	AIC	BIC	loglik	deviance	df.resid	ICC (cond.)
Model diagnostics								
Nullmodel	60,911	55	74,359.7	74,377.8	− 37,177.9	74,355.7	60,909	0.084
Model 2	60,911	55	54,111.8	54,391.3	− 27,024.9	54,049.8	60,880	0.087

*BCS* breast-conserving surgery, *MAS* mastectomy, *BCS-MAS* breast-conserving surgery followed by mastectomy, *cont* continuous, *ref* referent, *AIC* Akaike information criterion, *BIC* Bayesian information criterion, *loglik* log-likelihood, *df.resid* degrees of freedom residuals, *ICC* intraclass correlation coefficient

## Discussion

In the present study we analyzed data to describe the current clinical practice regarding NACT in 94,638 patients with early breast cancer in 55 breast cancer centers certified by the German Cancer Society (DKG) and the German Society of Senology (DGS). Patients were treated between 2007 and 2018. These centers were monitored regularly for their quality of breast cancer related structure and processes, diagnostic and treatment tools and results by annual site visits. They must fulfill criteria such as minimum numbers of patients treated, quality indicators, tumor boards, interdisciplinary teams and cancer registration (for details see: https://www.krebsgesellschaft.de/). Thus, clinical data analyzed here are generated by breast cancer centers with homogenous standards. The distribution of the centers included in this study represent the real-world clinical situation in Germany. Roughly, 80% of all breast cancer cases diagnosed in Germany are treated in certified centers (Annual Report 2020 of the Certified Breast Cancer Centres (BCCs). Audit year 2019/ indicator year 2020). It was shown that the use of NACT increased over time with a proportion of 5% in 2007 reaching levels of about 18% in 2017. In the same period the use of ACT decreased from 40 to 20%. By 2018 64% of patients did not receive any CHT at all compared with 55% in 2007. This development was similar in a study analyzing data provided to the West German Breast Center (WBC) by 105 breast cancer units (Riedel et al. 2020).

Male patients were treated less often with NACT in our analysis. As expected, patients with larger tumors, higher grading and with positive axillary lymph nodes received more often NACT. It is well known that NACT is more efficient in certain subtypes such as triple-negative or HER2-positive breast cancer. Current guidelines strongly recommend the use of NACT in these tumor types (Kommission Mamma der Arbeitsgemeinschaft Gynäkologische Onkologie e. V. in der Deutschen Gesellschaft für Gynäkolgie und Geburtshilfe e. V., sowie in der Deutschen Krebsgesellschaft e. V. 2020; Leitlinienprogramm Onkologie S3-Leitlinie Mammakarzinom 2021). When the optimal result of pCR is not achieved, patients may benefit from post-neoadjuvant treatments. In HR-positive, HER2-negative breast cancer NACT was only used in 5.8% of the patients whereas it was performed in 26.5% of patients with HR-positive and HER2-positive cancers (Table 2). A higher percentage of NACT of about 32% was observed in patients with triple-negative and HR-negative/HER2-positive cancer. Thus, ORs for NACT in these subtypes were 4.1 (95% CI 3.6–4.6) and 4.6 (95% CI 3.9–5.5) when compared to HR-positive, HER2-negative breast cancer. As expected, the pCR rates varied by subtype with a low rate of 12% in HR-positive, HER2-negative, higher rates of 36 and 38% in HR-positive, HER2-positive and triple-negative and the highest rate of 55% in HR-negative, HER2-positive cancers. The rate of pCR increased over time suggesting that more efficient treatments (e.g., drug and antibody combinations) were used in NACT regimes in recent years and selection of patients who benefit from these treatments improved. Similar observations were made in the WBC study mentioned above. In a recent meta-analysis from the Early Breast Cancer Trialist’s Collaborative Group (EBCTCG) the clinical complete response rate was 28% (Early Breast Cancer Trialists’ Collaborative Group (EBCTCG) 2018). The pCR rate was not published in this article but should be significantly lower than clinical response rate. In current clinical practice as shown in our study the choice of NACT or ACT is rather driven by the subtype than size of breast cancer. However, the ORs of NACT for primary breast cancer in stages T2 and 3 are 2.7 and 3.7. Thus, tumor size still is a factor that determines the use of NACT and also the ability of BCS after NACT. The increasing use of NACT with increasing tumor size is surprising in view of the fact that response rates of NACT are higher the smaller the tumor size. Clinical tumor stage is the most important predictor of pathological complete response rate after neoadjuvant chemotherapy in breast cancer patients (Goorts et al. 2017).

Our data demonstrate the current clinical practice of NACT in certificated breast cancer centers in Germany. According to the German National Cancer Plan these are networks of qualified and jointly certified interdisciplinary institutions that include the entire chain of health care for patients (Kowalski et al. 2017). Certified breast cancer centers must fulfill guideline-based criteria for treatment. Many of these criteria are specified as quality indicators (QI) which are measurable elements of practice performance and are part of the German S3 guideline for breast cancer (Leitlinienprogramm Onkologie S3-Leitlinie Mammakarzinom 2021). We recently reported that analyses of QI data are suitable to describe implementation of novel treatments and guideline adherence (Inwald et al. 2019). The tool OncoBox Research allows studies with the need for more detailed clinical information since it includes patient micro data.

Compared to other routinely collected data, the data used here come with a number of advantages. Compared to German claims data for example, our data are not selective regarding the insurance company and most importantly, they include information on clinical staging (Hoffmann and Glaeske 2010). Compared to the mandatory cancer registry data, OncoBox Research data has slightly higher completeness on clinical staging which is typically below 75% in breast cancer patients (Koch-Institut 2019). From a practical perspective, most striking is that the data are readily available in a uniform standard, with very high completeness and that they can be easily compiled across providers compared to mandatory registry data where analyses are often based on single or few regional registries (Inwald et al. 2017). When interpreting the results, however, some caution is required. Though data are partly quality-assured with sample checks during the on-site certification audits, they are not of the same high standard as clinical trial data. We especially expect some underreporting of treatments outside the operating site which includes ACT. We also expect learning effects among data collectors. Changes over time may be influenced by improved documentation in the centers. Compared to mandatory registries, our data were only collected in DKG/DGS certified units, leaving data of about 25% of patients treated in non-certified units unaccessible.

The use of routine practice data (sometimes referred to as “real world data”) for routine use is subject to ongoing national and international discussions (Schünemann 2019; Klinkhammer-Schalke et al. 2020). We suggest investing in research that compares strengths and weaknesses of different routine practice data sets to help researchers and readers to evaluate the quality of the data but also the strengths of the evidence they may generate.

This was the first study that analyzed quality assurance data from over 50 breast cancer centers using the OncoBox Research. Data were used to answer questions on quality of cancer care and clinical cancer research including changes over time. The use of NACT was introduced into clinical practice with increasing rates that differ depending on the subtype of breast cancer. Clinicians’ decisions are driven by their expectations on benefits of NACT. The resulting outcome parameter of pCR demonstrates increasing success of this strategy that was previously proven in randomized controlled trials.

## Data Availability

Not applicable.

## Appendix: Additional analyses

See Appendix Tables 6, 7, 8, 9.

A: Models including center effects

**Table 6:** Dependent variable NACT vs no NACT (Model 1b)

	Odds ratio	95% CI-lower	95% CI-upper
(Intercept)	0.00	0.00	8.22
Age (cont.)	0.93	0.93	0.94
Male (ref. female)	0.67	0.33	1.36
Year of diagnosis			
2007 (ref.)	1		
2008	1.38	0.91	2.08
2009	1.28	0.85	1.92
2010	1.37	0.92	2.06
2011	1.76	1.19	2.62
2012	1.93	1.33	2.82
2013	1.87	1.28	2.71
2014	1.87	1.29	2.70
2015	3.02	2.11	4.33
2016	4.69	3.28	6.70
2017	3.93	2.76	5.60
2018	3.40	2.38	4.86
Tumor size			
T0	1.17	0.36	3.82
TIS/DCIS	0.08	0.03	0.23
T1 (ref.)	1		
T2	2.69	2.43	2.99
T3	3.66	2.99	4.48
T4	6.31	5.11	7.80
Nodal status			
N0 (ref.)	1		
N1	3.28	2.95	3.65
N2	3.02	2.30	3.96
N3	2.28	1.51	3.45
Grading			
G1	0.25	0.19	0.33
G2 (ref.)	1		
G3	2.09	1.89	2.32
G4	0.70	0.16	3.08
Type of surgery			
Mastectomy	0.72	0.65	0.81
BCS (ref.)	1		
BCS+Mastectomy	0.41	0.32	0.51
Subtype			
HR+/HER− (ref.)	1		
HR −/HER −	4.07	3.58	4.64
HR+/HER+	4.12	3.62	4.68
HR −/HER+	4.59	3.86	5.46
Urbanity: more than 100,000 pop. (ref 100,000 or less)	7.32	0.17	318.09
University hospital (vs. not)	0.83	0.00	268.52
Ownership			
Private (ref.)	1		
Charitable	0.87	0.00	968.04
Public	2.03	0.00	2669.12
Primary cases 2018 (cont.)	1.00	0.98	1.02

Model	*N* patient	*N* center	AIC	BIC	loglik	deviance	df.resid	ICC (cond.)
Model diagnostics								
Nullmodel	65,667	55	18,919.2	18,937.4	− 9,457.6	18,915.2	65,665	0.920
Model 1	65,667	55	12,610.1	12,937.4	− 6,269.0	12,538.1	65,631	0.827

**Table 7:** Dependent variable ACT vs no CHT (Model 2b)

	Odds ratio	95% CI-lower	95% CI-upper
(Intercept)	55.13	23.15	131.30
Age (cont.)	0.93	0.93	0.93
Male (ref. female)	1.83	1.42	2.36
Year of diagnosis			
2007 (ref.)	1		
2008	1.00	0.88	1.15
2009	1.05	0.93	1.19
2010	0.90	0.80	1.03
2011	0.79	0.69	0.89
2012	0.67	0.59	0.76
2013	0.61	0.54	0.69
2014	0.60	0.53	0.68
2015	0.54	0.48	0.61
2016	0.49	0.43	0.55
2017	0.54	0.47	0.61
2018	0.44	0.39	0.50
Tumor size			
T0	0.96	0.63	1.48
TIS/DCIS	0.59	0.48	0.73
T1 (ref.)	1		
T2	1.68	1.60	1.77
T3	1.48	1.31	1.66
T4	0.73	0.63	0.84
Nodal status			
N0 (ref.)	1		
N1	3.50	3.31	3.70
N2	5.11	4.54	5.75
N3	4.83	4.11	5.67
Grading			
G1	0.28	0.26	0.30
G2 (ref.)	1		
G3	2.77	2.63	2.93
G4	0.58	0.18	1.85
Type of surgery			
Mastectomy	0.72	0.68	0.77
BCS (ref.)	1		
BCS+Mastectomy	1.26	1.15	1.37
Subtype			
HR+/HER− (ref.)	1		
HR−/HER−	3.11	2.86	3.37
HR+/HER+	3.73	3.45	4.03
HR−/HER+	3.54	3.14	3.98
Urbanity: more than 100,000 pop. (ref 100,000 or less)	0.61	0.40	0.95
University hospital (vs. not)	1.36	0.71	2.62
Ownership			
Private (ref.)	1		
Charitable	0.71	0.31	1.62
Public	0.48	0.21	1.11
Primary cases 2018 (cont.)	1.00	1.00	1.00

Model	*N* patient	*N* center	AIC	BIC	loglik	deviance	df.resid	ICC (cond.)
Model diagnostics								
Nullmodel	60,911	55	74,359.7	74377.8	− 37,177.9	74,355.7	60,909	0.084
Model 2	60,911	55	54,114.3	54,438.9	− 27,021.1	54,042.3	60,875	0.075

B Sensitivity analyses

B1. Models with separate effects for missing information

**Table 8:** Dependent variable NACT vs no NACT (Model 1c)

	Odds ratio	95% CI-lower	95% CI-upper
(Intercept)	0.19	0.11	0.34
Age (cont.)	0.93	0.93	0.93
Male (ref. female)	0.34	0.18	0.64
Year of diagnosis			
2007 (ref.)	1		
2008	1.51	1.17	1.96
2009	0.88	0.68	1.14
2010	0.78	0.60	1.01
2011	1.53	1.19	1.96
2012	1.52	1.18	1.95
2013	1.57	1.23	2.02
2014	1.73	1.35	2.21
2015	2.56	2.02	3.26
2016	4.26	3.36	5.39
2017	3.87	3.07	4.90
2018	3.60	2.84	4.55
Tumor size			
T0	0.35	0.12	1.02
TIS/DCIS	0.01	0.00	0.02
T1 (ref.)	1		
T2	2.65	2.43	2.90
T3	3.51	2.96	4.15
T4	5.66	4.74	6.77
Missing	0.01	0.01	0.02
Nodal status			
N0 (ref.)	1		
N1	2.93	2.69	3.19
N2	2.47	2.06	2.97
N3	1.75	1.33	2.29
Missing	5.23	3.45	7.92
Grading			
G1	0.26	0.20	0.34
G2 (ref.)	1		
G3	2.12	1.94	2.33
G4	0.85	0.20	3.57
Missing	1432.42	1215.72	1687.74
Type of surgery			
Mastectomy	0.90	0.82	0.98
BCS (ref.)	1		
BCS+Mastectomy	0.47	0.39	0.56
Subtype			
HR+/HER− (ref.)	1		
HR− /HER −	3.98	3.59	4.41
HR+/HER+	3.86	3.47	4.28
HR− /HER+	4.43	3.85	5.09
Missing	0.31	0.24	0.41

Model	*N* patient	*N* center	AIC	BIC	loglik	deviance	df.resid	ICC (cond.)
Model diagnostics								
Nullmodel	94,638	55	63,414.8	63,433.7	− 31,705.4	63,410.8	94,636	0.093
Model 1	94,638	55	20,683.9	21.014.9	− 10,306.9	20,613.9	94,603	0.175

**Table 9:** Dependent variable ACT vs no CHT (Model 2c)

	Odds ratio	95% CI-lower	95% CI-upper
(Intercept)	23.76	18.67	30.25
Age (cont.)	0.93	0.93	0.93
Male (ref. female)	1.69	1.36	2.10
Year of diagnosis			
2007 (ref.)	1		
2008	1.10	0.99	1.22
2009	1.05	0.94	1.16
2010	0.98	0.88	1.08
2011	0.88	0.79	0.97
2012	0.76	0.69	0.85
2013	0.69	0.62	0.77
2014	0.65	0.59	0.73
2015	0.59	0.53	0.66
2016	0.56	0.50	0.62
2017	0.58	0.53	0.65
2018	0.48	0.44	0.54
Tumor size			
T0	0.85	0.56	1.30
TIS/DCIS	0.54	0.44	0.66
T1 (ref.)	1		
T2	1.61	1.54	1.69
T3	1.29	1.15	1.45
T4	0.63	0.55	0.72
Missing	0.87	0.80	0.95
Nodal status			
N0 (ref.)	1		
N1	3.82	3.64	4.00
N2	6.38	5.81	7.01
N3	5.57	4.92	6.30
Missing	0.68	0.53	0.87
Grading			
G1	0.26	0.24	0.28
G2 (ref.)	1		
G3	2.85	2.72	2.99
G4	0.50	0.22	1.17
Missing	0.72	0.62	0.83
Type of surgery			
Mastectomy	0.77	0.73	0.81
BCS (ref.)	1		
BCS+Mastectomy	1.31	1.21	1.41
Subtype			
HR+/HER− (ref.)	1		
HR −/HER−	3.36	3.13	3.60
HR+/HER+	3.88	3.63	4.15
HR −/HER+	3.67	3.31	4.06
Missing	0.67	0.59	0.76

Model	*N* patient	*N* center	AIC	BIC	loglik	deviance	df.resid	ICC (cond.)
Model diagnostics								
Nullmodel	83,860	55	101,933.4	101,952.1	− 50,964.7	101,929.4	83,858	0.080
Model 2	83,860	55	73,177.4	73,504.2	− 36,553.7	73,107.4	83,825	0.152
